# The Emerging Role of Probiotics and their Derivatives against Biofilm-Producing MRSA: A Scoping Review

**DOI:** 10.1155/2022/4959487

**Published:** 2022-12-27

**Authors:** Saba Jalalifar, Rasoul Mirzaei, Tahereh Motallebirad, Shabnam Razavi, Malihe Talebi

**Affiliations:** ^1^Microbial Biotechnology Research Center, Iran University of Medical Sciences, Tehran, Iran; ^2^Department of Microbiology, School of Medicine, Iran University of Medical Sciences, Tehran, Iran; ^3^Venom and Biotherapeutics Molecules Lab, Medical Biotechnology Department, Biotechnology Research Center, Pasteur Institute of Iran, Tehran, Iran; ^4^Department of Microbiology, School of Medicine, Isfahan University of Medical Sciences, Isfahan, Iran

## Abstract

**Background:**

Methicillin-resistant *Staphylococcus aureus* (MRSA) is one of the main bacterial pathogens causing chronic infections, mainly because of its capacity to produce biofilm. Biofilm production is one of the underlying strategies for antibacterial drug resistance. Accordingly, preventing and attenuating biofilm production has become an emerging approach to controlling persistent infections. Therefore, this scoping review is aimed at surveying the published literature describing the usage of probiotics and their derivatives against biofilm-producing MRSA.

**Methods:**

Updated literature searches were conducted across seven electronic databases including Web of Science, PubMed, Scopus, Cochrane Library, ProQuest, Embase, and Google Scholar to identify all original published articles about probiotics against MRSA. In this regard, studies were summarized and analyzed in the present review.

**Results:**

In the reviewed studies, various microorganisms and compounds were used as probiotics as follows: *Lactobacillus* species (8 studies), *Enterococcus* species (4 studies), *Bacillus* species (2 studies), *Streptomyces* species (2 studies), *Saccharomyces cerevisiae* (1 study), *Corynebacterium accolens* (1 study), and *Lactococcus lactis* derived Nisin (3 studies). Based on our comprehensive search, 21 studies with eligibility criteria were included in the present review including 12 studies on clinical strains, 6 studies on ATCC, 2 studies simultaneously on clinical and standard strains, and finally 1 study on food sample.

**Conclusions:**

Our study showed that there was an increasing trend in the number of publications reporting probiotics against biofilm-producing MRSA. The results of this scoping review could use to guide the undertaking of the subsequent systematic reviews. In summary, probiotics with antimicrobial and antibiofilm properties can use as an embedded agent in food products or as a biopharmaceutical in the prevention and treatment of MRSA infections.

## 1. Introduction

**Figure 1:**
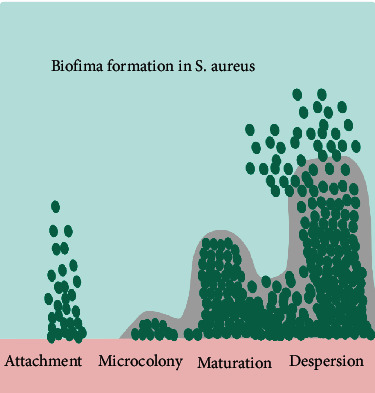
Biofilm formation in *S. aureus*.

Biofilm is a sessile community of bacteria embedded in a self-produced extracellular polymeric matrix attached to a substratum and it is generally composed of extracellular DNA (eDNA), proteins, and polysaccharides, which is very compatible with unfavorable environmental conditions [1–4]. Biofilm is associated with more than 65% of all bacterial infections [5–7]. In the 1970s, Bill Costerton found a link between the cause of persistent infection and bacterial accumulation in patients with cystic fibrosis, resulting in the introduction of a community mode of growth so-called biofilm [8, 9]. The stages of the biofilm formation include (i) attachment of planktonic bacteria to a surface or each other, (ii) formation of microcolonies and extracellular polymeric substances, (iii) maturation of the biofilm, and (iv) dispersal of the biofilm-embedded bacteria (Figure 1) [10, 11]. In some cases, the biofilm formed by probiotic bacteria is potentially active against the development of infections by pathogenic bacteria [12]. On the other hand, biofilm produced by pathogenic bacteria causes infection in humans [13–16]. The pathogenetic role of biofilm, particularly in chronic infections, has been documented because of its hijacking ability of immune system, and resistance to antibiotics [8, 17]. Biofilm is problematic because of its drug-resistant capacity and ability to evade the mechanisms of human defense, which hinder infection treatment [7, 18, 19]. Bacterial biofilm formation happens in planktonic cells because of environmental switches and contributes to the transfer of genes from one microorganism to another under various environmental stress [7, 18].

**Figure 2:**
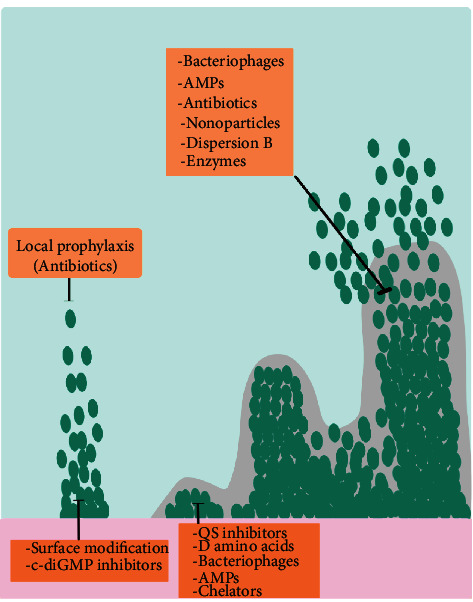
Some principal strategies against bacterial biofilm.

*Staphylococcus aureus* is one of the most important biofilm-forming pathogen with a wide variety of complications as well as life-threatening infections [20]. In this regard, Methicillin-resistant *S. aureus* (MRSA) is one of the most successful strains and is transmitted in both healthcare and community settings resulting in skin and soft tissue infections, bone infections, joint infections, bacteremia, and endocarditis, among others [21, 22]. The rapid and increasing development of antibiotic resistance, especially in *S. aureus*, has become a serious concern [21]. According to reports, up to 11,000 cases in USA die annually from MRSA-related infections, which represents almost half of all deaths from antibiotic-resistant bacteria [23–25]. Even with the ongoing development of new antibiotics, active surveillance efforts, and advances in infection prevention, MRSA remains a prominent pathogen with persistently high mortality [26]. The World Health Organization (WHO) recently published a list of priority pathogenic bacteria such as MRSA that urgently needs new antibiotics [21, 27, 28]. Most importantly, with the emergence of biofilm-forming multidrug-resistant (MDR) *S. aureus* strains, the need for more effective therapeutic approaches is essential [29, 30]. Some principal strategies have been developed to interrupt biofilm formation in the distinct stages of development, such as inhibition of bacterial adhesion, destruction of preformed biofilm, and the use of quorum-quenching agents that inhibit quorum sensing, among others (Figure 2) [31]. However, these approaches are not completely effective, and considering the increasing resistance of MDR-MRSA strains and their tendency to form biofilms, it has been suggested that their eradication should not depend on mentioned strategies alone [32–34].

Probiotics are usually defined as live microbial cells that when administered in adequate amounts, confer a health benefit on the host [35]. Evidence shows that probiotic strains can act as adjuncts to antibiotic therapy by reducing adverse effects, improving antibiotic function, and enhancing mucosal immunity [36]. Probiotic bacteria play a significant role in preventing or treating gastrointestinal infections in humans [37]. The secretion of antimicrobial compounds including organic acids such as short-chain fatty acids (SCFAs) has been a well-documented attribute of probiotic bacteria [36, 38]. Probiotics also have a protective role, directly competing with pathogens through signaling interference [39]. Probiotic-derived mediators such as lactic acid, hydrogen peroxide, and bacteriocins have been found to be effective against bacterial pathogen growth, adhesion, and biofilm formation [36]. Besides, since MRSA resides in the normal microflora, it could not be eliminated easily with antibiotics; hence, probiotics and their derivatives to prevent and eliminate pathogenic biofilms are more rational [34]. In this regard, the use of probiotic strains such as Lactic acid bacteria (LAB) was found to be an eradication option against biofilms [40, 41]. The most well-known probiotic bacteria such as LAB, *Bifidobacteria*, *Bacillus coagulans*, and *Saccharomyces boulardii* have been reported [42, 43]. Our goal in this scoping review was to describe and discuss the role of probiotics and their derivatives on biofilm-producing MRSA.

## 2. Methods

### 2.1. Search Strategy

International databanks, including Web of Science, PubMed, Scopus, Cochrane Library, ProQuest, Embase, and Google Scholar, were searched from November 8, 2020 to June 7, 2021. In the present study, Mesh, EMtree, and the free text method were used to determine synonyms by the following keywords: (Biofilm OR “Biofilm Matrix” OR “Biofilm Matrices” OR (Matrix AND Biofilm) OR “EPS Matrix” OR “EPS Matrices” OR (Matrix AND EPS) OR “Extracellular Polymeric Substances” OR (“Polymeric Substance” AND Extracellular) OR Exopolymer OR (Matrix AND Extracellular) OR “Extracellular Matrices” OR (Matrices AND Extracellular) OR “Bacterial Polysaccharides” OR (Polysaccharides AND Bacterial) AND Probiotic AND 1996/01/01 : 2021/03/31[dp]).

### 2.2. Study Selection and Data Extraction

The records found through database searching were merged, and the duplicates were removed using EndNote X8 (Thomson Reuters, New York, NY, USA). Two reviewers (Saba Jalalifar and Tahereh Motallebirad) independently screened the records by title and abstract to exclude those not related to the aim of the current study. The full texts of potentially eligible records were retrieved and evaluated. Besides, selected articles were peer-reviewed and the extracted data were organized based on the authors' names, published time, location, source of MRSA, probiotics, source of probiotics, probiotic components, and the outcomes.

#### 2.2.1. Inclusion Criteria and Exclusion Criteria

- All original and experimental studies related to biofilm, probiotics, and MRSA were included. Besides, clinical trial studies, nonclinical trial studies, and animal experiments were also included
- The reviews, meta-analyses, systematic reviews, case reports, and correspondences were excluded from our study

Besides, studies with insufficient information and Congress abstracts were also excluded.

This scoping review used a thematic analysis to compare studies and identify them for further research because the topic spans disciplines that depend on both qualitative and quantitative research, and because many of the included studies relied on various probiotic species, MRSA, and small sample sizes. The complete Stages of the Scoping Review Framework are depicted in Table 1.

## 3. Results

### 3.1. Database Search and Characterization of Studies

**Figure 3:**
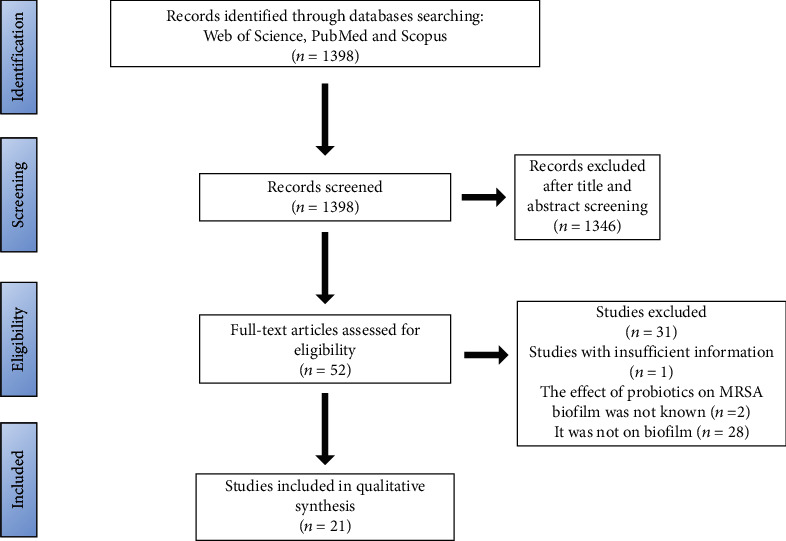
The flow of the evidence selection in the present review.

In total, 1398 records were identified via database searching, in which after screening 1346 records were excluded by title and abstract checking. In the next step, in 52 remained records based on our comprehensive search, 21 studies with eligibility criteria were included in the present review. In brief, 12 studies were conducted in Asian countries, and most of the studies on this continent are related to India (3 studies). Among these 21 studies, 12 cases were on clinical strains, 6 cases on ATCC, 2 cases on clinical and standard isolates simultaneously, and finally 1 case on food samples. The flow chart of the evidence selection in the present review is shown in Figure 3. The complete characteristics of the included studies are depicted in Table 2.

### 3.2. Type of Probiotics

In the reviewed studies, various microorganisms and compounds were used as probiotics as follows: *Lactobacillus* species (8 studies), *Enterococcus* species (4 studies), *Bacillus* species (2 studies), *Streptomyces species* (2 studies), *Saccharomyces cerevisiae* (1 study), *Corynebacterium* accolens (1 study), and *Lactococcus lactis* derived Nisin (3 studies).

In all studies that used *Lactobacillus* species*, Enterococcus* species, *Streptomyces* species, *L. lactis*, *Saccharomyces cerevisiae*, and *Corynebacterium accolens* as a probiotic compound, a decrease in biofilm formation was observed. Also, the antibiofilm effect of Nisin was observed in 3 studies. One study using *Bacillus subtilis* and *Bacillus amyloliquefaciens* observed an inhibitory effect against the biofilm-associated MRSA and methicillin-susceptible *S. aureus* (MSSA).

### 3.3. Dose of Probiotics

In some studies, the dose of administrative probiotics was noted. For example, in a study using MRSAcin as a probiotic, the inhibitory concentration against MRSA biofilm was 125 *μ*g/mL [44]. In another study, the inhibitory effect of different concentrations of antimicrobial compounds produced by members of the genus *Bacillus* (AMC) on the biofilm formation of MRSA was determined. In current study, the total biofilm formation estimated by crystal violet staining showed a significant decrease via a dose dependent manner of AMC as follows: 0.5 *μ*g: 0.23 ± 0.01; 1 *μ*g: 0.06 ± 0.01; 4 *μ*g: 0.05 ± 0.001, and 1 mg: 0.07 ± 0.001 compared to the control (1.08 ± 0.01) [45]. Besides, in a study conducted by Mohamed et al. [46] in Saudi Arabia and Egypt, 50 and 100 mg/mL of *Lactobacilli biosurfactants* were used to inhibit the biofilm formation of MRSA. Compared to the control, MRSA biofilm was inhibited by *L. biosurfactants* at mentioned concentrations for 18 h. In another study, the antibiofilm effect of two species of *Lactobacillus* against MRSA was determined. In current study, biosurfactants of *L. jensenii* and *L. rhamnosus* showed antibiofilm activities against *S. aureus* at 25-50 mg/mL [47]. Finally, in a study from Iran, the antibiofilm effect of the methanolic extract of *Streptomyces* sp. MUSC 125a against MRSA was found at 1.5625 mg/mL [48].

## 4. Discussion

The antibiotic resistance of *S. aureus* has become a major public health concern, and MRSA strains are one of the most frequent causes of nosocomial infections worldwide [21]. On the other hand, biofilm formation by *S. aureus* could add another problem to its antibiotic resistance phenotype, resulting in serious and persistent infections [49]. Effective antibiofilm agents are required to interrupt and damage biofilm-associated pathogens. In this regard, probiotics can prevent colonization as well as biofilm of pathogens at the site of infection, and compete with them for nutrients showing an interesting application toward the infection [50].

In a study by Braïek et al. [51], two strains of *Enterococcus lactis* named Q1 and 4CP3 were used as probiotics to inhibit the biofilm formation of MRSA. Cell-Free Supernatant (CFS) from *E. lactis* Q1 and 4CP3 displayed antibiofilm capacities with a highly synergistic binary combination. In two other studies in Spain and France [52, 53], the antibiofilm effect of *E. faecalis* was evaluated. The first study found that enterocin DD28 and DD93 improve the inactivation of planktonic and sessile *Staphylococci* and reduce their biofilm formation in combination with a certain biocide [52]. In the other study, Gómez et al. [53] found that bacteriocins (enterocin AS-48—purified from the cultures supernatants of *E. faecalis*) were able to synergize with erythromycin and kanamycin, two antibiotics used in the MRSA treatment. Also, a study conducted by Boopathi et al. [54] in India examined the inhibitory effect of *E. durans* and found that CFS of bacteria significantly reduced biofilm formation in MRSA (94 ± 0.9%). Therefore, the *Enterococcus* species can be proposed with other antibiotics to treat MRSA infections and must be more attention [54].

Many studies used *Lactobacillus* as a probiotic, indicating the high importance of these bacteria [33, 46, 55]. For example, a study in Turkey examined the antibiofilm effect of CFS on four different species of *Lactobacillus* (*L. acidophilus*, *L. plantarum*, *L. fermentum*, and *L. rhamnosus*) and foundall tested CFSs inhibit biofilm formation significantly (*P* < 0.0001) [55]. Kumar et al. [33] investigated the antibacterial effect of LAB biofilm isolated from Tairu and Kefir against MRSA biofilm. In thier study, *L. casei* and *L. plantarum* were used and found a decrease in the formation of MRSA biofilm, but *L. casei* showed better inhibitory potential against MRSA [33]. Also, another study by Mohamed et al. [46] found that *L. biosurfactants* inhibited MRSA biofilm at 50 and 100 mg/mL for 18 h. Additionally, the antibiofilm activity of *L. biosurfactants* as promising medications against MRSA infections in animals was reported. In two other studies in South Korea [56] and Turkey [57], lipoteichoic acid (LTA) and cell-free extract (CFE) of *L. plantarum* were used to inhibit MRSA biofilm formation, respectively. In these studies, an antibiofilm effect was found against MRSA biofilm. In 2017, Kang et al. [58] used CFS of *L. salivarius* and *L. fermentum*, isolated from the oral mucosa of healthy children (4–7 years), to inhibit biofilm formation. The results showed that *L. salivarius* had a strong bactericidal effect against MRSA biofilm. In contrast, *L. fermentum* did not affect *S. aureus* biofilm cells [58]. In a study conducted by Sambanthamoorthy et al. [47] *L. jensenii* and *L. rhamnosus* showed antimicrobial and antibiofilm activities against *S. aureus*. Also, a study performed in the USA showed that the supernatant of *L. rhamnosus* effectively reduces MRSA biofilm [59]. Therefore, according to these studies, most *Lactobacillus* species have a significant antibiofilm effect against MRSA, so these bacteria can be used to further investigation as a treatment against *S. aureus* infections.

*Bacillus* species are another kind of probiotic bacteria that have been studied to inhibit biofilm formation. In two studies, these bacteria were used as a probiotic. In the study conducted by Algburi et al. [60], the combinations of cefotaxime with *B. subtilis* and *B. amyloliquefaciens* were used, and the CFS of bacilli strains showed an inhibitory effect against MRSA and MSSA biofilm. These findings confirmed the ability of beneficial bacteria to compete with the pathogens at the site of colonization or for the nutrient source. The current study found no significant differences in the biofilm prevention activity of CFS of *B. subtilis* and *B. amyloliquefaciens* against MRSA and MSSA. Another study noted that a certain strain of *B. paralicheniformis* (UBBLi30) can produce the antimicrobial peptide bacitracin with biological activity against a range of gram-positive bacteria and inhibition of MRSA biofilm [45]. Accordingly, *Bacillus* species can also be used as probiotics to treat *S. aureus* infections. However, it is suggested that more studies be done on these bacteria and their antibiofilm effects.

In a study performed by Singh and Dubey [61], a new strain of endophytic *Actinobacterium* was isolated from the plant *Datura metesl*, which produced secondary metabolites with potent anti-infective activities. Based on *16S rRNA* gene sequence analysis, this isolate was identified as *Streptomyces californicus* strain ADR1. ADR1 derived metabolites were able to effectively inhibit the formation of biofilm of MRSA strains by up to 90% reduction at a significantly lower concentration of the metabolites [61]. Also *Streptomyces* sp. strain MUSC 125 from Mangrove soil in Malaysia was found using *16S rRNA* phylogenetic analysis and the methanolic extract of this strain showed antibiofilm, anti-MRSA, and antioxidant activities [48]. Overall, these studies show the potential of *Streptomyces* strains as a promising source of antibiofilm and anti-MRSA compounds that warrant more attention and research.

In a study conducted by Saidi et al. [62] from Iran, supernatant and lysate extracts of yeast *S. cerevisiae* isolated from sweet fruit samples were used to inhibit the formation of MRSA and MSSA biofilm. They found that both extracts have reduced the biofilm formation of MRSA and the MRSA strain showed more susceptibility to yeast extracts than the MSSA strain in all tests [62]. The current study found suitable antagonistic effects of *S. cerevisiae* as a probiotic on MRSA and MSSA strains. Accordingly, the compounds produced by this yeast can be further evaluated to determine its control ability against *S. aureus* infections, and more similar studies should be performed to confirm these findings.

In the study of Menberu et al. [63], the antibiofilm potential of *C. accolens* CFS on *S. aureus,* and MRSA biofilms was assessed. The supernatants of *C. accolens* induced a significant reduction in metabolic activity and biofilm biomass of *S. aureus* and MRSA clinical isolates compared to untreated growth control (*P* < 0.05). In this investigation, *C. accolens* demonstrated antibacterial activity against *S. aureus* and MRSA clinical isolates in both planktonic and biofilm forms, suggesting the potential creation of novel probiotic medicines to enhance sinus health.

One group of compounds with enormous potential for therapeutic application is lantibiotics (bacterially derived antimicrobial peptides) [64]. Lantibiotics are ribosomally synthesized peptides that are defined by the presence of unusual amino acids, including lanthionine and/or methyllanthionine [65]. The most meticulously investigated lantibiotic is Nisin produced by *L. lactis.* Nisin has a antibacterial activity against a wide range of gram-positive bacteria, including foodborne pathogens such as Staphylococci, Clostridia, and Bacilli [66]. In a study, Field et al. [67] examined the antibiofilm effect of nisin and they found a significant decrease in the metabolic activity of established biofilms *S. aureus* treated with nisin V + chloramphenicol and nisin I4V + chloramphenicol combinations showed. In another study, Muunim et al. [44] investigated, and compared the effects of purified MRSAcin (new bacteriocins from MRSA), Nisin, and vancomycin on MRSA biofilm and they found that purified MRSAcin at 125 *μ*g/mL was more effective on MRSA biofilm. This study suggested that the effect of pure MRSAcin against MRSA biofilm is more than Nisin and vancomycin at different concentrations. The tested bacteriocins showed the highest bactericidal activation agent MRSA biofilm material and suggest that bacteriocin from MRSA attacks biofilm cells more effectively than vancomycin, although is widely used in first-line therapy for different MRSA infections. These results show that bacteriocins can be raised as a good alternative candidate for antibiotics in the treatment of drug-resistant bacterial infections. In a study in South Africa, Ahire and Dicks [68] investigated the antibiofilm effect of Nisin incorporated with 2, 3-Dihydroxybenzoic Acid (DHBA) in Nanofibers against MRSA. They found that biofilm formation decreased by 88% after 24 h of exposure to Nanofibers containing Nisin and DHBA, compared to a 63% decrease when exposed to Nanofibers containing only DHBA and a 3% decrease when exposed to Nanofibers containing only Nisin [68]. Taken together, these results showed that Nisin has a better antibiofilm effect when used with DHBA than when used alone.

## 5. Limitations

One of the limitations of the studies included in this review was that most studies have not quantitatively investigated the inhibitory effect of probiotics on biofilm formation and have reported only qualitative results. Also, in many studies, the concentration of probiotics to inhibit biofilm formation was not mentioned. As a result, it is impossible to conclude exactly what dose of the probiotics has an antibiofilm effect. Considering that the purpose of investigating the antibiofilm effect of these probiotics is to use them as drugs for the treatment of patients, therefore, it is important to know their effective dose. The next limitation was the difference in the biofilm formation ability of strains because it has been found that various strains are different in terms of biofilm formation ability and resistance to antimicrobial agents, which make the results variable [69, 70]. On the other hand, in these studies, various techniques have been used to investigate the reduction or inhibition of biofilm formation, which causes heterogeneity of results. For example, in some studies, the microtiter plate test was used, while in other studies, cell culture or spot-on-lawn method/spot-on-agar method was used. Besides, the methodological quality of included studies varied from weak to moderate. Some studies were faced with selecting a small study sample and different sizes, and different methodological approaches. Finally, the other limitation of this study was limited to the English language for searches that missed some interesting data.

## 6. Conclusion

A growing body of documents shows that when given in sufficient quantities for an extended period, probiotics are beneficial in some diseases and safer than some drugs. In terms of infectious diseases, these probiotic bacteria and their compounds show antimicrobial and antibiofilm properties against MRSA. It should be noticed that data are still scarce and there is not enough evidence to consider probiotics as biodrugs to inhibit pathogenic biofilm formation bacteria and/or disperse preformed biofilms. Future investigations are needed to further determine the best probiotic and dose for specific infections, first, in the animal models as well as in clinical trials. Besides, insights regarding precise mechanisms of probiotics and their derivatives against biofilm infections are essential to be determined. In summary, in the future, these probiotics can be used as embedded in food products or biodrugs in the treatment of bacterial infections. This is important, especially in the treatment of drug-resistant bacteria such as MRSA, and can be a suitable alternative to antibiotics.

## Figures and Tables

**Table 1:** Scoping review framework.

Stage	Stage name	Description
Stage 1	The research question for appropriate search	The emerging role of probiotics and their derivatives against biofilm-producing MRSA.
Stage 2	Identifying relevant studies	Records identified through databases searching: web of science, PubMed, Scopus, Cochrane library, ProQuest, Embase, and Google scholar.
Stage 3	Study selection	Records excluded after title and abstract screening.
Stage 4	Eligibility	Eligibility was done based on inclusion and exclusion criteria mentioned in the text.
Stage 5	Charting the data	Present in the text and the second figure.
Stage 6	Collating, summarizing, and reporting the results	Present in the text .

**Table 2:** Overview on probiotics used against MRSA.

	First author	Published time	Location	Source of MRSA	Probiotics/dose	Source of probiotics	Probiotic components	Outcomes	References
1	Laavanya M. Kumar	2017	Malaysia	ATCC	Lactic acid bacteria (LAB)	Tairu and kefir	Biofilm	Reduction of MRSA biofilms	[33]
2	Ben Braïek Olfa	2018	Tunisia	ATCC	*Enterococcus lactis*	Raw white and pink shrimps	Cell-free supernatants (CFSs)	Reduced of biofilm	[51]
3	Fatma Kalaycı Yüksek	2020	Turkey	ATCC	Four diferent *lactobacillus* species' (*L. acidophilus*, *L. plantarum*, *L. fermentum* and *L. rhamnosus*	ATCC	CFSs	All tested CFSs were shown to inhibit biofilm formation significantly (*P* < 0.0001).	[55]
4	Ammar Algburi	2020	Iraq	Clinical isolates	*Bacillus subtilis* and *Bacillus amyloliquefaciens*	Unknown	CFSs	Inhibitory effect against biofilm-associated MRSA and MSSA	[60]
5	Natacha Caballero Gómez	2013	Spain	Clinical isolates	*Enterococcus faecalis*	*Enterococcus faecalis*	Enterocin	Enterocin can improve the inactivation of planktonic as well as sessile staphylococci in combination with a certain biocide	[53]
6	Hind H. Muunim	2019	Iraq	Food samples	Nisin 125 *μ*g/mL		Vancomycin and Nisin and MRSAcin	Purified MRSAcin at 125 *μ*g/mL more affected against MRSA biofilm than vancomycin and Nisin	[44]
7	J. J. Ahire	2020	India	ATCC	*Bacillus paralicheniformis* 0.5 *μ*g/mL, 1 *μ*g/mL, 4 *μ*g/mL, and 1 mg/mL	Traditional fermented food.	CFSs	The CFS of bacilli strains showed an inhibitory effect against MRSA and MSSA biofilm	[45]
8	Jayesh J. Ahire	2015	South Africa	Clinical isolates	Nisin		Nisin	Biofilm formation decreased by 88% after 24 h of exposure to nanofibers containing Nisin and DHBA (NDF), compared to a 63% decrease when exposed to nanofibers containing only DHBA (DF) and a 3% decrease when exposed to nanofibers containing only Nisin (NF)	[68]
9	Seenivasan Boopathi	2017	India	Clinical isolates	*Enterococcus durans*	Unpasteurized cow's milk sample	Supernatant	Significantly reduced biofilm formation in MRSA (94 ± 0.9%)	[54]
10	Essam Hassan Mohamed	2020	Saudi Arabia/Egypt	Clinical isolates	*Lactobacillus biosurfactants* 50 and 100 mg/mL	Yogurt	Biosurfactants	The antibiofilm activity of *Lactobacillus biosurfactants* as promising medications for the treatment of *S. aureus* MRSA in animals.	[46]
11	Des Field	2016	Ireland	Clinical isolates	Nisin	*Lactococcus lactis*	Nisin	The metabolic activity of established biofilms treated with Nisin V + chloramphenicol and Nisin I4V + chloramphenicol combinations revealed a significant decrease in *S. aureus*	[67]
12	Ahmed K. Al Atya	2016	France	Clinical isolates	*Enterococcus faecalis*	Meconium (the dark green substance forming the first feces of a newborn infant.)	CFSs	These bacteriocins were able to synergize with erythromycin and kanamycin, two antibiotics used in the MRSA treatment.	[52]
13	Ki Bum Ahn	2018	South Korea	Clinical isolates	*Lactobacillus plantarum*	The Korean collection for type culture (Daejeon, Korea)	Lipoteichoic acid (LTA)	Inhibitory effect	[56]
14	Mi-sun Kang	2017	Switzerland	Clinical isolates	*Lactobacillus salivarius* and *Lactobacillus fermentum*	The oral mucosa of healthy children (4–7 years).	CFSs	*L. salivarius* had a strong bactericidal effect against biofilm *S. aureus*. In contrast, *L. fermentum* had no effect on *S. aureus* biofilm cells	[58]
15	Navid Saidi	2019	Iran	ATCC	*Saccharomyces cerevisiae*	Sweet fruit samples	Supernatant and lysate extracts	Both extracts have reduced biofilm formation. The MRSA strain showed more susceptibility to yeast extracts than the MSSA strain in all tests.	[62]
16	Karthik Sambanthamoorthy	2014	USA	Clinical isolates	*Lactobacillus jensenii* and *Lactobacillus rhamnosus* 25-50 mg/mL	ATCC	Biosurfactants	Both *L. jensenii* and *L. rhamnosus* showed antibiofilm and antimicrobial activities against *S. aureus.*	[47]
17	Radha Singh	2020	India	ATCC/clinical isolates	*Streptomyces californicus*	Plant, Datura metel	ADR1 metabolites	ADR1 metabolites were able to effectively inhibit the formation of biofilm by the *S. aureus* and the MRSA strains. Up to 90% reduction in the formation of biofilm could be achieved at a significantly lower concentration of the metabolites.	[61]
18	Hefa Mangzira Kemung	2020	Malaysia	ATCC	*Streptomyces* sp. 1.5625 mg/mL	Mangrove soil in Malaysia	Methanolic extract	The methanolic extract of strain MUSC 125 showed antibiofilm, anti-MRSA, and antioxidant activities	[48]
19	Tugce Onbas	2019	Turkey	ATCC/clinical isolates	*Lactobacillus plantarum*	The fecal microbiota of healthy breastfed infant	Cell-free extract (CFE)	Inhibit MRSA biofilm formation	[57]
20	Martha Alemayehu Menberu	2021	Australia	Clinical isolates	*Corynebacterium accolens*	Predominant species of the healthy human nasal microbiota	CFSs	*C. Accolens* supernatants induced a significant reduction in metabolic activity and biofilm biomass of *S. aureus* and MRSA clinical isolates compared to untreated growth control (*P* < 0.05).	[63]
21	Yi Wang	2018	USA	Clinical isolates	*Lactobacillus rhamnosus*	Commercial probiotic drink	Supernatant	Effectively reduces biofilm	[59]

## Data Availability

Data available on request from the authors.
